# Jingfukang induces anti-cancer activity through oxidative stress-mediated DNA damage in circulating human lung cancer cells

**DOI:** 10.1186/s12906-019-2601-x

**Published:** 2019-08-07

**Authors:** Zujun Que, Zhiyi Zhou, Bin Luo, Changsheng Dong, Yi Jiang, Hegen Li, Jianhui Tian

**Affiliations:** 1 Oncology Institute of Traditional Chinese Medicine, Shanghai Research Institute of Traditional Chinese Medicine, No. 725, South Wanping Road, Shanghai, 200032 China; 2 Department of Oncology, Longhua Hospital, Shanghai University of Traditional Chinese Medicine, No. 725, South Wanping Road, Shanghai, 200032 China

**Keywords:** Non-small cell lung cancer, Circulating tumor cell, Jinfukang, Apoptosis, Oxidative stress

## Abstract

**Background:**

Metastasis is the main cause of lung cancer death. As a seed of metastasis, circulating tumor cells are an important target for metastasis intervention. The traditional Chinese medicine, Jinfukang, has been clinically available for the treatment of non-small cell lung cancer (NSCLC). In this study, we investigated the action and underlying mechanisms of Jinfukang against circulating lung tumor cells.

**Methods:**

The cell counting kit-8 (CCK-8), colony formation and cell cycle assays were used to study the cell proliferation ability. Flow cytometry was used to detect the apoptosis and the expression level of ROS and Caspase-3. Comet and TUNEL assays were used to detect DNA damage. DNA damage related pathway protein was detected by western blot.

**Results:**

Jinfukang significantly inhibits the proliferation of CTC-TJH-01 cells by inducing G1 phase arrest and inhibits their colony formation in a dose-dependent manner. Moreover, Jinfukang induces apoptosis in CTC-TJH-01 cells through the ROS-mediated ATM/ATR-p53 pathway and DNA damage.

**Conclusions:**

Our findings suggest that Jinfukang may be a potential drug for lung cancer metastasis.

**Electronic supplementary material:**

The online version of this article (10.1186/s12906-019-2601-x) contains supplementary material, which is available to authorized users.

## Background

Lung cancer is the leading cause of cancer-associated deaths, 85% of which is non-small cell lung cancer (NSCLC) [1]. Current multidisciplinary treatment for lung cancer can reduce disease recurrence and increase long-term survival of the patients [2, 3]. However, existing therapies have limited benefits for lung cancer patients at the early stage. Numerous studies have shown that adjuvant chemotherapy, targeted therapy and immunotherapy do not improve the prognosis and survival of patients with early-stage lung cancer and cause serious side effects and complications [4–6]. The main reason is that current chemotherapy and targeted therapy are based on the design of treatment options for primary lung cancer, but for early lung cancer patients, there is a lack of anti-metastatic drugs [7].

Circulating tumor cells (CTCs) represent the primary cause of intractable metastatic disease and are considered essential for metastasis formation. The latest research shows that the number of CTCs is closely related to the metastasis of lung cancer [8]. A guideline for the clinical practice of malignancy issued by the National Comprehensive Cancer Network (NCCN) has incorporated CTCs into the TNM (tumor node metastasis) staging system [9]. In addition, the eighth edition of the cancer staging system developed by the American Joint Committee on Cancer (AJCC) lists CTCs in the peripheral blood as a prognostic factor of breast cancer [10]. Hashimoto M and his colleagues found that increasing pvCTC count was significantly correlated with postoperative distant metastasis in completely resected NSCLC patients [11]. Therefore, targeting CTCs to develop specific anti-metastatic drugs may be the key to improving the clinical efficacy of the early-stage lung cancer. The current research and development of lung cancer drugs mainly focuses on the tissues and cells of primary tumors, which is the main reason for leading to poor clinical efficacy.

Traditional Chinese Medicine (TCM) has been used to treat for treatment various diseases. Jinfukang oral liquid, a Chinese herbal prescription, consists of 12 Chinese herbal medicines (Additional file 1: Table S1) and was approved by the State Food and Drug Administration in 1996 (Z19991043). In clinical practice, Jinfukang is specifically used in the treatment of NSCLC, which has been proved to be capable of preventing the occurrence of metastasis, stabilizing tumor lesions, improving the response rates when combined with chemotherapy, and prolonging the survival period of lung cancer patients [12, 13]. However, the clinical effects of Jinfukang are certain, but the biological mechanism is unclear.

In this study, we examined the effects of Jinfukang on a circulating human lung cancer tumor cell line and studied the underlying molecular mechanisms involved, which might provide experimental evidence for clinical therapy in lung cancer.

## Methods

### Chemicals and reagents

Jinfukang oral liquid freeze-dried powder was prepared and detected the fingerprint (Additional file 2: Table S2) by Professor Yu Jin of East China University of Science and Technology (Shanghai, China). A cell counting kit-8 (CCK-8) was obtained from Dojindo. The Annexin V-FITC apoptosis detection kit and propidium iodide (PI) were purchased from BD Pharmingen. 2,7-Dichlorodihydrofluorescein diacetate (DCFH-DA) and the antioxidant NAC were purchased from Sigma. A caspase-3 detection kit was purchased from Biovision. The anti-γ-H2AX, anti-p-ATM, anti-p-ATR, anti-PARP1, goat anti-mouse IgG-HRP and donkey anti-rabbit IgG-HRP antibodies were purchased from Cell Signaling Technology. The anti-p53, anti-p21, anti-CDK4, anti-Cyclin D, anti-Cyclin E, anti-Fas, anti-Survivin, and anti-β-actin antibodies were purchased from Proteintech.

### Cell culture

The circulating human lung cancer cell line CTC-TJH-01 was isolated from the peripheral blood of patients with stage II lung cancer [14]. The cells were cultured in F12K medium (Gibco, USA) with 10% fetal bovine serum (Biological Industries, Israel) and incubated at 37 °C in a humidified air containing 5% CO_2_.

### Cell viability assay

The cells were seeded into 96-well plates at a density of 3 × 10^3^ per well and allowed to grow overnight. Jinfukang was dissolved in DMSO and diluted with F12K medium to the final concentrations of 125, 250, 500, and 1000 μg/mL. The tumor cells were incubated with Jinfukang for 24, 48 and 72 h before the CCK-8 assay (Dojindo, Japan). Absorbance values were measured at 450 nm and survival curves were plotted using GraphPad software.

### Colony forming assay

The CTC-TJH-01 cells were seeded in 6-well plates (300 cells/well), allowed to attach overnight, and then treated with Jinfukang (350 or 700 μg/mL) for 10 days. The cells were fixation with 4% polyoxymethylene, washed thrice with PBS, and then stained with Giemsa for 30 min. The cells were then scanned with an HP scanner, and each well was counted.

### Cell cycle analysis and detection of caspase-3 activation

For the cell cycle analysis, the cells were stained with PI and analyzed by a FACSVerse™ flow cytometer (BD Biosciences, CA) as described previously [15]. Caspase-3 activation was determined using a CaspGLOW™ fluorescein active caspase-3 staining kit (Biovision, Mountain View, CA).

### Cell apoptosis analysis

The cells were treated with Jinfukang (350 or 700 μg/mL) for 48 h, and then collect the cells, washed twice with ice-cold PBS, and evaluated for apoptosis by double stains with FITC-conjugated annexin V and PI in binding buffer for 30 min using a FACSVerse flow cytometer.

### Measurement of ROS generation

Cellular ROS contents were measured by flow cytometry as described previously [15]. Briefly, the cells were treated with Jinfukang (350 or 700 μg/mL) for 48 h, and stained with 10 μM DCFH-DA (Sigma, USA) at 37 °C for 30 min. The cells were collected and the fluorescence was analyzed using a FACSVerse flow cytometer.

### TUNEL assay

The cells were treated with Jinfukang (350 or 700 μg/mL) for 48 h, and then examined for apoptosis using the in situ cell death detection kit (Roche, China) according to the manufacturer’s instruction manual.

### Immunofluorescence staining

The cells were treated with Jinfukang (350 or 700 μg/mL) for 48 h, fixed with 4% paraformaldehyde for 30 min, incubated with 0.3% Triton X-100 for 10 min, and blocked with 0.1% BSA in PBS for 1 h. The primary anti-γ-H2AX antibody (1:200, CST, USA) was added and incubated overnight at 4 °C, and the cells were incubated for 1 h with the appropriate secondary antibody. The slides were incubated with 0.5 mg/ml DAPI (Sigma, USA) for 5 min. All images were observed under a confocal microscope equipped with a digital camera (Leica, Germany).

### Comet assay

The Comet assay was performed as previously described [16]. The DNA damage of CTC-TJH-01 cells was detected by single cell gel electrophoresis assay. For analysis of the comet images, the extent of DNA damage was estimated by fluorescence microscopy using the semiquantitative method of visual scoring.

### Western blot analysis

Western blotting was conducted as described previously [17]. In brief, the cell lysates and proteins were extracted. 40 μg of the proteins was used to detect. The immunoreactive bands were visualized by using an ECL kit (Bio-Rad, Hercules, CA). The density of the immunoreactive bands was analyzed using ImageJ computer software (National Institutes of Health, MD).

### Statistical analysis

All experiments were assayed in triplicate (*n* = 3). The data are expressed as the mean ± SEM. All statistical analyses were performed using GraphPad Prism 6.0 (GraphPad, SanDiego, CA). The differences in the measured variables between the experimental and control groups were assessed by the Student’s test. *P* < 0.05 was defined as statistically significant.

## Results

### Jinfukang inhibits proliferation of CTC-TJH-01 cells

**Fig. 1:**
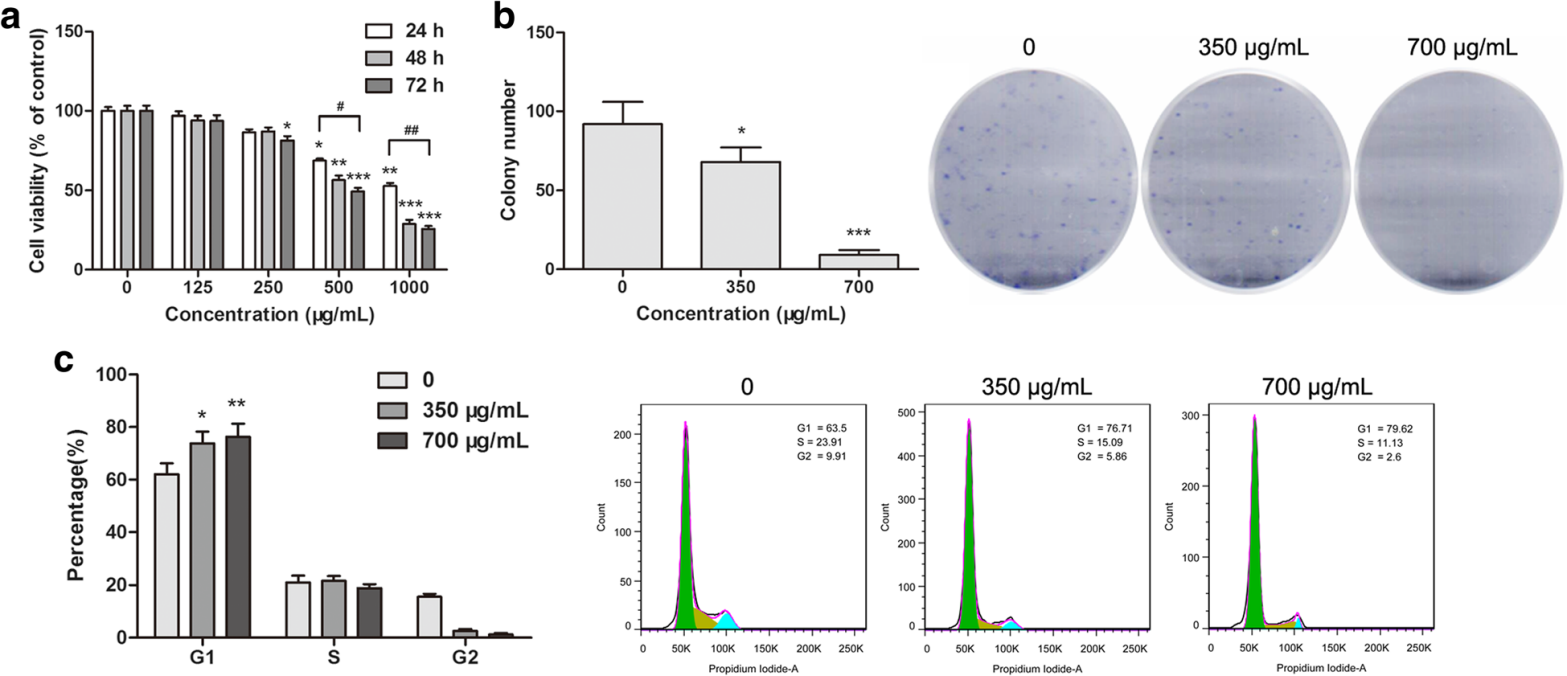
Jinfukang inhibits CTC-TJH-01 cell growth. **a** CTC-TJH-01 cells were incubated with Jinfukang (0, 125, 250, 500, and 1000 μg/mL) for 24 h, 48 h and 72 h. The CCK-8 assay was performed to determine the cytotoxic effect of Jinfukang. **b** Representative images of the colony formation assay. **c** CTC-TJH-01 cells were treated with Jinfukang (0, 350, and 700 μg/mL) for 48 h. Flow cytometry was performed to determine the cell cycle. Each bar represents the means±SD of three separate experiments. *P < 0.05; **P < 0.01; ***P < 0.001

The cytotoxicity of Jinfukang to CTC-TJH-01 cells was measured by the CCK-8 assay. We found that Jinfukang decreased the cell viability of the CTC-TJH-01 cells in a time- and dose-dependent manner (Fig. 1a). The IC_50_ of Jinfukang at 48 h was 728.8 ± 4.4 μg/mL. In addition, Jinfukang also can inhibited the number of clones of the CTC-TJH-01 cells in a dose-dependent manner (Fig. 1b), and arresting the CTC-TJH-01 cells in the G1 phase (Fig. 1c). These data suggested that Jinfukang exhibits potent cytotoxicity against the CTC-TJH-01 cells.

### Jinfukang induces apoptosis in CTC-TJH-01 cells

**Fig. 2:**
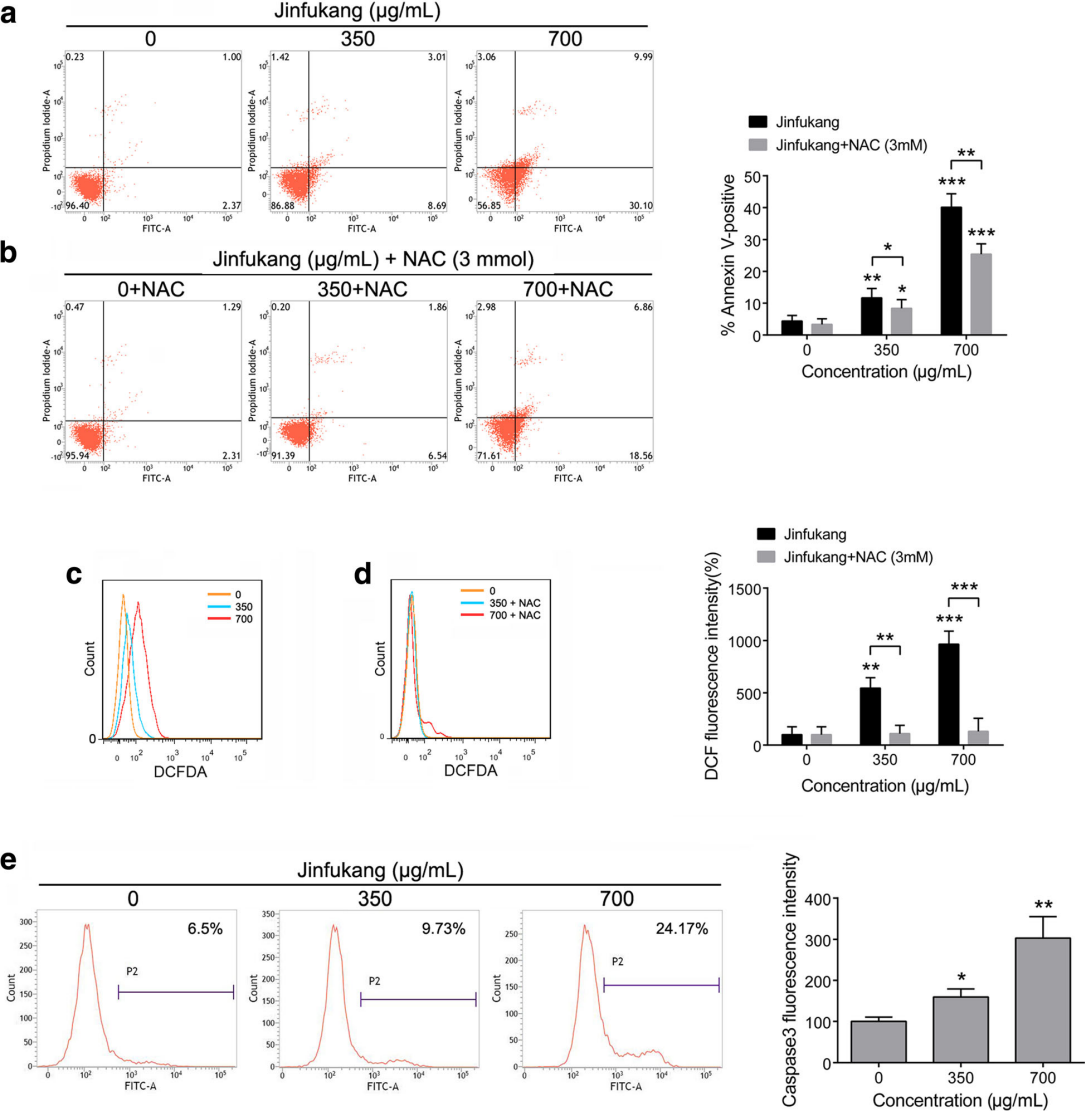
Jinfukang-induced apoptosis in CTC-TJH-01 cells. **a** and **b** CTC-TJH-01 cells were treated with Jinfukang (0, 350, and 700 μg/mL) or Jinfukang cotreatment with NAC for 48 h. Flow cytometry was performed to determine the apoptosis of CTC-TJH-01 cells. **c** and **d** Flow cytometry was performed to detection the ROS level in the CTC-TJH-01 cells after treatment with Jinfukang. **e** Flow cytometry was performed to determine the level of caspase-3 in the CTC-TJH-01 cells. Each bar represents the mean ± SD of three separate experiments. *P < 0.05; **P < 0.01; ***P < 0.001

We used annexin V/PI staining assay to detect the apoptosis of CTC-TJH-01 cells. It was found that Jinfukang can significantly induce apoptosis in CTC-TJH-01 cells. (Fig. 2a). In addition, we also determined the levels of caspase-3 and ROS in CTC-TJH-01 cells. The results show that Jinfukang can increase the levels of caspase-3 and ROS (Fig. 2c, e). The above results suggest that Jinfukang-induced apoptosis of CTC-TJH-01 cells may be through ROS pathway activation.

### Jinfukang can induce CTC-TJH-01 cells apoptosis through ROS pathway

To further clarify the mechanism of Jinfukang inducing apoptosis of CTC-TJH-01 cells, the antioxidant N-acetyl cysteine (NAC, 3 mM) was used in the treatment. It was found that Jinfukang-mediated ROS induction was inhibited (Fig. 2d). Furthermore, we also found that NAC attenuated CTC-TJH-01 cells apoptosis (Fig. 2b). These results proved that Jinfukang-induced growth inhibition and apoptosis of CTC-TJH-01 cells through ROS pathway activation.

### Jinfukang can induce DNA damage in CTC-TJH-01 cells

**Fig. 3:**
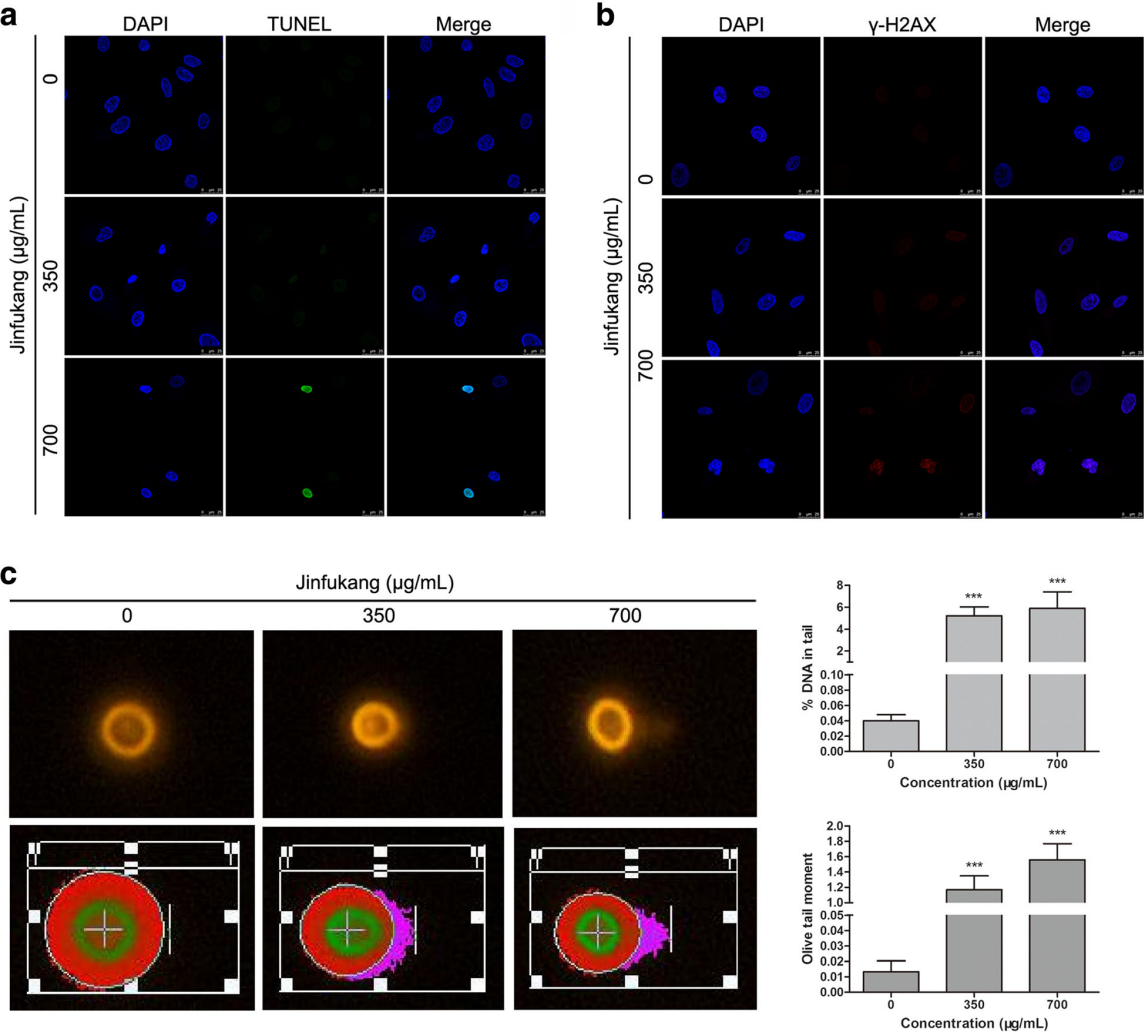
Jinfukang-induced DNA damage in CTC-TJH-01 cells. **a** and **b** CTC-TJH-01 cells were treated with Jinfukang (0, 350, and 700 μg/mL) for 48 h. Immunofluorescence was used to detect DNA damage and γ-H2AX expression in the CTC-TJH-01 cells. **c** Detection of nuclear damage in the CTC-TJH-01 cells by comet electrophoresis. Each bar represents the mean ± SD of three separate experiments. *P < 0.05; **P < 0.01; ***P < 0.001

Then, we detected the DNA integrity of CTC-TJH-01 cells. TUNEL detection results show that the DNA damage was increased in the CTC-TJH-01 cells after treatment with Jinfukang (Fig. 3a). Furthermore, we also found that Jinfukang significantly upregulated the expression of γ-H2AX protein in the nucleus of CTC-TJH-01 cells (Fig. 3b). These results suggested that Jinfukang may promote the apoptosis of CTC-TJH-01 cells by inducing DNA damage. Hence, the comet assay was also used to confirm that Jinfukang can induce DNA damage in CTC-TJH-01 cells. These results indicate that Jinfukang induce the apoptosis of CTC-TJH-01 cells through ROS-mediated oxidative DNA damage.

### Jinfukang induces the apoptosis of CTC-TJH-01 cells through the ROS-mediated ATM/ATR-p53 pathway

**Fig. 4:**
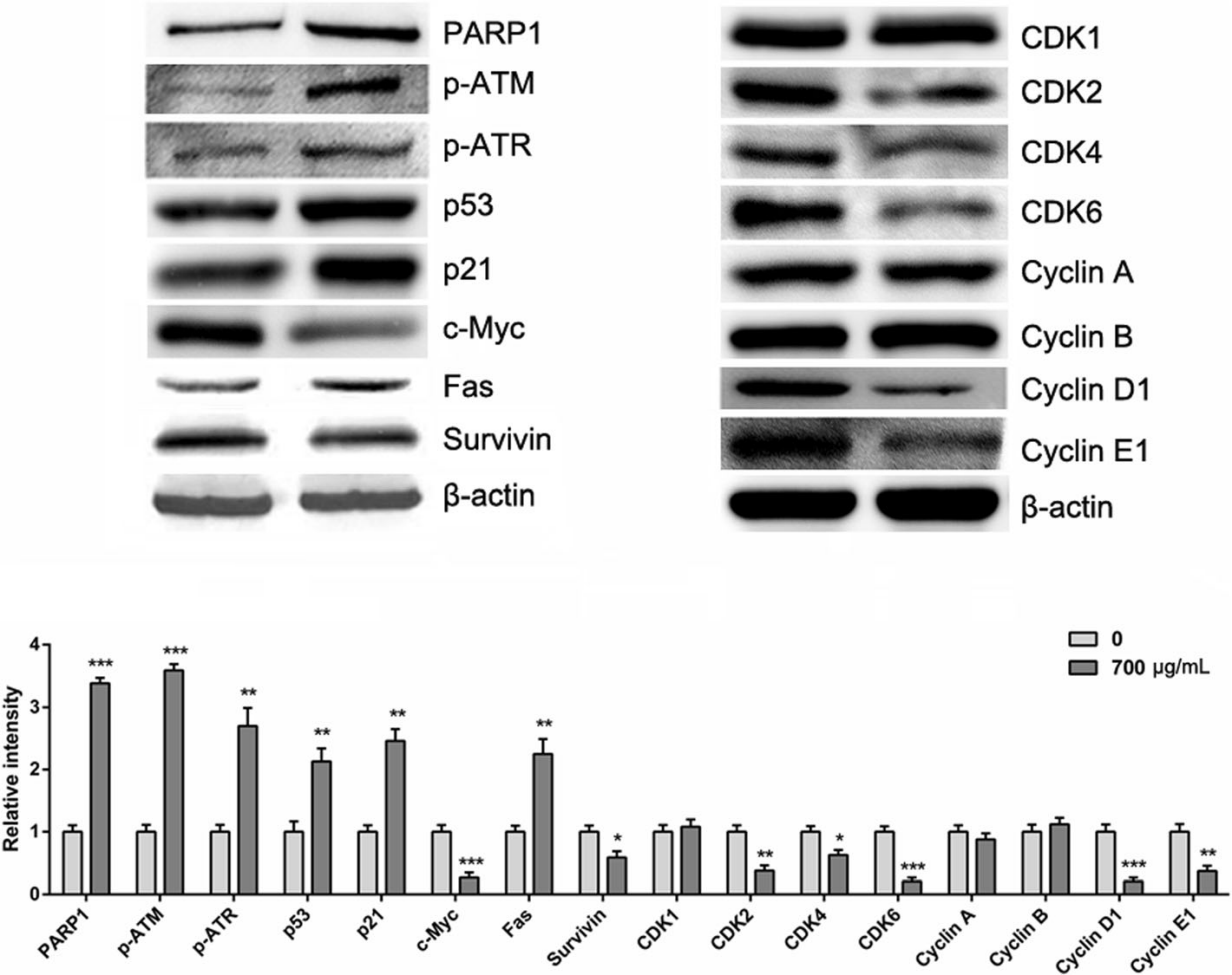
Apoptosis related protein expression was detected by western blot analysis after treatment of the CTC-TJH-01 cells with Jinfukang (0, 350, 700 μg/mL) for 48 h. β-actin was used as an internal standard

**Fig. 5:**
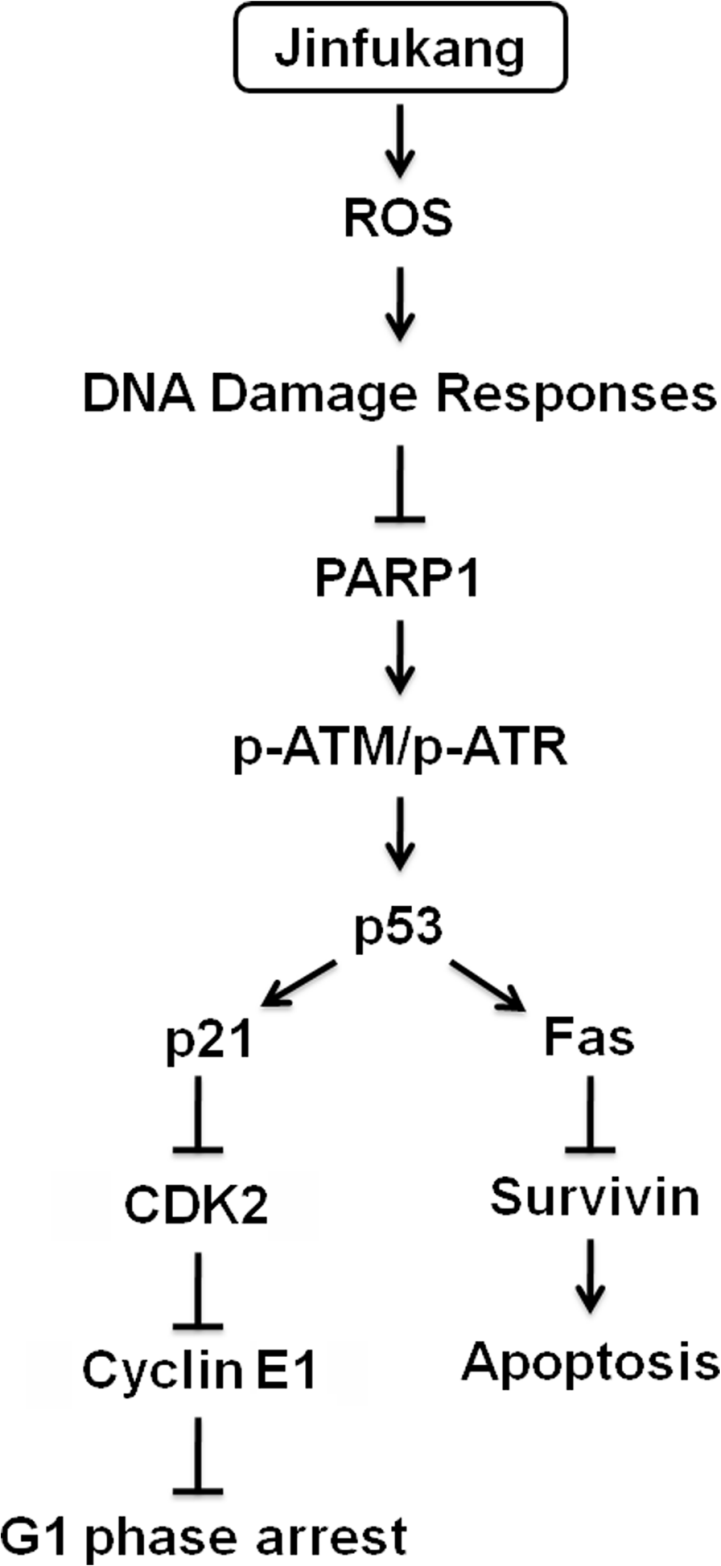
A pattern diagram of Jinfukang-induced apoptosis in lung adenocarcinoma

To further reveal the mechanism of Jinfukang inducing CTC-TJH-01 apoptosis, we studied the ROS-mediated DNA damage-related ATM/ATR-p53 pathways. It was found that the expression of phospho-ATM, phospho-ATR, PARP1, p53, p21 and Fas proteins was significantly upregulated when the CTC-TJH-01 cells were treated with Jinfukang, and the expression of surviving protein was down-regulated (Fig. 4). In addition, Jinfukang inhibited the expression of the CDK2, CDK4, CDK6, cyclin D1 and cyclin E1 proteins in the CTC-TJH-01 cells (Fig. 4). Taken together, these results indicated that Jinfukang can induce DNA damage in CTC-TJH-01 cells through the ROS-mediated ATM/ATR-p53 signaling pathway, and the proliferation of the CTC-TJH-01 cells is arrested in the G1 phase through the p53-p21 signaling pathway (Fig. 5).

## Discussion

It is well accepted that metastasis is the main cause of lung cancer death, and prevention of postoperative metastasis is an urgent need for current lung cancer treatment [18]. However, metastasis remains the most poorly understood component of cancer pathogenesis, and most existing drugs inhibit only cancer cell proliferation. There are two complementary anti-metastasis strategies, the prevention of cancer cell dissemination and the suppression of metastases already in existence [7]. The theory of “seed and soil” suggests that circulating tumor cells may be the seeds disseminated by the cancer cells. Recent studies have shown that the number of CTCs is associated with the metastasis and survival of patients with lung cancer [8]. Our previous study found that CTC-TJH-01 cells are in the intermediate stage of EMT transformation, with strong drug resistance, stem cell phenotype and immune escape characteristics. In vivo studies have shown that CTC-TJH-01 cells have tumorigenicity and lung metastasis ability [19]. Therefore, targeting CTCs may be the key to preventing metastasis and improving the survival of patients with lung cancer.

Chinese herbal medicine has been increasingly used for the treatment of cancer. The TCM formula, Jingfukang, has been used for more than 20 years to treat lung cancer in clinical practice. Clinical results have confirmed that Jinfukang can prevent the recurrence and metastasis of lung cancer and prolong the survival of patients [20]. However, the cellular and molecular mechanisms remain unclear. Previous studies have demonstrated that Jingfukang can promote DDP-induced apoptosis in lung cancer cells [21, 22]. In addition, other studies have also found that Jinfukang can inhibit lymphatic endothelial cell formation and migration and inhibit the lung tumor mass via the suppression of bone marrow-derived mesenchymal stem cell transformation and lung tumor lymphangiogenesis [23, 24].

Based on the above results, we speculate that Jinfukang may inhibit the recurrence and metastasis of lung cancer by interfering with CTCs in the peripheral blood of patients with lung cancer. Therefore, we studied the intervention effect of Jinfukang on the CTC-TJH-01 cells. The results showed that Jinfukang significantly inhibited the proliferation of the CTC-TJH-01 cells, arrested their proliferation in the G1 phase, and inhibited their monoclonal-forming ability. We found that Jinfukang triggered G1 phase arrest in CTC-TJH-01 cells by up-regulating the expression of p53 and p21 levels and down-regulating the expression levels of CDK2, CDK4, CDK6, Cyclin D1, and Cyclin E1. These results are consistent with previous reports showing that p21 can induce G1 phase arrest through suppress Cyclin D1/CDK4 complex [25]. Further study found that Jinfukang significantly induced apoptosis in CTC-TJH-01 cells. These findings are in agreement with previous reports in that Jinfukang can induce apoptosis and cell cycle progression in lung adenocarcinoma cancer cells [21, 22].

The present study demonstrates that Jinfukang can induce the apoptosis of lung cancer cells by activating the expression of AIFM2 [21]. In addition, Jinfukang can also induce apoptosis of lung adenocarcinoma cells through p53 and MAPK signaling pathways [21, 22]. Nevertheless, it was not found that Jinfukang induced apoptosis in lung cancer cells by intervening ROS pathway. Previous studies have confirmed that increased ROS levels can cause DNA damage and induce cell apoptosis [26]. Hence, we conducted research on this pathway and found that Jinfukang can significantly increase the levels of ROS in CTC-TJH-01 cells. Furthermore, Jinfukang-induced increase in ROS and cell death can partly reversed when cotreatment with NAC. Our study also found that Jinfukang can induce DNA damage in CTC-TJH-01 cells. DNA damage response mainly involves signal pathways such as ATM/ATR-p53, p38/MAPK-Atf2 and BMP-Smad1 [27]. Our study found that Jinfukang can increase the expression of PARP1, p-ATM, p-ATR, p53 and p21 after treatment of the CTC-TJH-01 cells. Taken together, these results indicate that Jinfukang-induced apoptosis in CTC-TJH-01 cells through the ROS-mediated ATM/ATR-p53 pathway and DNA damage.

## Conclusions

In this study, we have demonstrated that Jinfukang induces cell cycle arrest and ROS-dependent apoptosis in circulating lung cancer cells. Furthermore, we affirmed that Jinfukang induces DNA damage through ROS and the upregulation of p-ATM, p-ATR, p53 and p21. These results provide an insight into the anti-CTC activities of Jinfukang and provide the basis for its clinical application.

## Additional files

Additional file 1: Jinfukang Ingredients. (DOCX 16 kb)

Additional file 2: Jinfukang fingerprint. (DOC 156 kb)

## Data Availability

All data generated or analysed during this study are included in this published article (and its additional information files).

## Abbreviations

AJCC: American Joint Committee on Cancer
CTCs: Circulating tumor cells
DCFH-DA: 2,7-Dichlorodihydrofluorescein diacetate
NAC: N-Acetyl-L-cysteine
NSCLC: Non-small cell lung cancer
PI: Propidium iodide
TNM: Tumor node metastasis
TUNEL: TdT-mediated dUTP Nick-End Labeling
