# Supplementary material for: Jingfukang induces anti-cancer activity through oxidative stress-mediated DNA damage in circulating human lung cancer cells
Source: BMC Complement Altern Med. 2019 Aug 7;19:204. doi: 10.1186/s12906-019-2601-x (PMC6686466; doi:10.1186/s12906-019-2601-x)
Supplement: Supplementary file 1 — Jinfukang Ingredients. (DOCX 16 kb) [file 12906_2019_2601_MOESM1_ESM.docx]

**Additional file 1: Table S1** Jinfukang components

| Latin name | Chinese name | Weight (g) |
| --- | --- | --- |
| Astragalus membranaceus | Huang Qi (黄芪) | 30 |
| Glehnia littoralis | Bei Sha Shen (北沙参) | 30 |
| Asparagus cochinchinensis | Tian Men Dong (天冬) | 10 |
| Ligustrum lucidum | Nv Zhen Zi (女贞子) | 10 |
| Selaginella doederleinii | Shi Shang Bai (石上柏) | 30 |
| Paris polyphylla | Chong Lou (重楼) | 15 |
| Epimedium sagittatum | Yin Yang Huo (淫羊藿) | 10 |
| Gynostemma pentaphyllum | Jiao Gu Lan (绞股蓝) | 10 |
| Cornus officinalis | Shan Zhu Yu (山茱萸) | 10 |
| Salvia chinensis | Shi Jian Chuan (石见穿) | 30 |
| Ophiopogon japonicus | Mai Dong (麦冬) | 10 |
| Trigonella foenum graecum | Hu Lu Ba (葫芦巴) | 10 |
