# Supplementary material for: Jingfukang induces anti-cancer activity through oxidative stress-mediated DNA damage in circulating human lung cancer cells
Source: BMC Complement Altern Med. 2019 Aug 7;19:204. doi: 10.1186/s12906-019-2601-x (PMC6686466; doi:10.1186/s12906-019-2601-x)
Supplement: Supplementary file 2 — Jinfukang fingerprint. (DOC 156 kb) [file 12906_2019_2601_MOESM2_ESM.doc]

HPLC condition: A Unitary C18 (4.6 mm × 150 mm, 5 μm, Acchrom) column was used for RPLC analysis. The column temperature was set at 25 ^o^C. Injection volumes were 5 μL. The flow rate was 1.0 mL/min. Mobile phase A was H_2_O (0.1% TFA) and B was ACN(0.1% TFA). Gradient was as following: 0-5 min, A/B, (95/5, v/v), 5-55 min, A/B, (95/5, v/v) → (60/40, v/v). The detecting wavelength was set at 254 nm.

B

A

C

D

E

F

G

H

I

J

K

L

M

A. Jin Fukang

B黄芪*Astragalus membranaceus* C. 石上柏*Selaginella doederleinii Hieron*

D. 石见穿*Salvia chinensis* E.绞股蓝*Gynostemma pentaphyllum*

F. 重楼*Paris polyphylla*  G. 女贞子*Ligustrum lucidum*

H. 麦冬*Ophiopogon japonicus* I. 天冬*Asparagus cochinchinensis*

J. 北沙参*Glehnia littoralis* K. 淫羊藿*Epimedium brevicornu*

L. 葫芦巴*Trigonella foenum-graecum* L. M.山茱萸*Cornus officinalis*

The results of the relevant standard comparison test are as follows:

The detecting wavelength was set at 203 nm.

1. Paris saponin VII

**Jinfukang**

**46.817**

**47.430**

**48.098**

**54.287**

**55.128**

**55.697**

**56.720**

**AU**

**0.00**

**0.10**

**0.20**

**0.30**

**0.40**

**0.50**

**0.60**

**0.70**

**Minutes**

**0.00**

**10.00**

**20.00**

**30.00**

**40.00**

**50.00**

**60.00**

**0.80**

2. β-​D-​Glucopyranoside, (3β,​25R)​-​17-​hydroxyspirost-​5-​en-​3-​yl O-​6-​deoxy-​α-​L-​mannopyranosyl-​(1→2)​-​O-​[α-​D-​glucopyranosyl-​(1→3)​]

3. Paris H

4. Dioscin

5. Gracillin

6. Polyphyllin I

7. Paris saponin V
